# Service evaluation of diabetes management during pregnancy in a regional maternity hospital: potential scope for increased self-management and remote patient monitoring through mHealth solutions

**DOI:** 10.1186/s12913-019-4471-9

**Published:** 2019-09-13

**Authors:** Abdelrahim Alqudah, Paul McMullan, Anna Todd, Conor O’Doherty, Anne McVey, Mae McConnell, John O’Donoghue, Joe Gallagher, Chris J. Watson, Lana McClements

**Affiliations:** 1 The Wellcome-Wolfson Institute for Experimental Medicine, School of Medicine, Dentistry and Biomedical Sciences, Queen’s University Belfast, Belfast, United Kingdom; 2 School of Pharmacy, The Hashemite University, Zarqa, Jordan; 3 Craigavon Area Hospital, Southern Health and Social Care Trust, Craigavon, United Kingdom; 4 ASSERT Centre, University College Cork, Cork, Ireland; 5 gHealth Research group, School of Medicine, University College Dublin, Dublin 4, Ireland; 6 School of Life Sciences, Faculty of Science, University of Technology Sydney, Sydney, Australia

**Keywords:** Pregnancy, Diabetes, Gestational diabetes, Type 1 diabetes, Type 2 diabetes, Antenatal clinic, Home monitoring, Digital technology

## Abstract

**Background:**

Pre-gestational and gestational diabetes mellitus are common complications in pregnancy affecting one in six pregnancies. The maternity services are under significant strain managing the increasing number of complex pregnancies. This has an impact on patients’ experience of antenatal care. Therefore, there is a clear need to address pregnancy care. One possible solution is to use home-based digital technology to reduce clinic visits and improve clinical monitoring.

**Methods:**

The aim of this study was to evaluate the antenatal services provided to pregnant women with diabetes who were monitored at the joint metabolic and obstetric clinic at the Southern Health and Social Care Trust in Northern Ireland.

**Results:**

The questionnaires were completed by sixty-three women, most of whom had gestational diabetes mellitus. Most of the participants were between 25 and 35 years of age (69.8%), had one or more children (65.1%) and spent over 2 h attending the clinics (63.9%); 78% of women indicated that their travel time to and from the clinic appointment was over 15 min. Over 70% of women used smartphones for health-related purposes. However, only 8.8% used smartphones to manage their health or diabetes. Less than 25% of the women surveyed expressed concerns about using digital technology from home to monitor various aspects of their health in pregnancy.

**Conclusions:**

Overall, pregnant women who had or developed diabetes in pregnancy experience frequent hospital visits and long waiting times in the maternity clinics. Most of these pregnant women are willing to self-manage their condition from home and to be monitored remotely by the healthcare staff.

## Background

Hyperglycaemia is the most common complication in pregnancy. It affects one in six pregnancies and it can lead to a number of complications such as miscarriage, still birth, pre-eclampsia, obstructed labour, increased risk of caesarean section, pre-term birth and the development of type 2 diabetes (T2D) in later life [1–3]. Diabetes in pregnancy includes pre-gestational diabetes, namely type 1 diabetes mellitus (T1DM) or T2DM, and gestational diabetes mellitus (GDM). GDM is defined as glucose intolerance with onset and first recognition in pregnancy [4]. The latest guidelines on the diagnosis of GDM provided by the World Health Organization (WHO, 2013) include the following parameters: fasting blood glucose over 5.1 mmol/l (91.8 mg/dl), 1-h postprandial blood glucose above 10 mmol/l (180 mg/dl) and 2-h postprandial blood glucose of 8.5 mmol/l (153 mg/dl) [5]. However, there are notable differences between individual hospitals and countries in screening and diagnosis of GDM [6].

Globally, over 21 million women are affected by some form of hyperglycaemia in pregnancy; GDM accounting for over 85% of these cases [7]. The vast majority of these cases are in low and middle-income countries with limited access to antenatal care. The increased incidence of diabetes in pregnancy has also negatively affected the healthcare services globally, which are struggling to meet the demand of high and increased number of complex pregnancies. Telehealth solutions have been proposed to relieve the pressure on both pregnant women and the health care service providers. However, to date, these services offer no advantage over standard care [8, 9].

In this study, we carried out a service evaluation at the Southern Health and Social Care (HSC) Trust, one of five Trusts providing integrated health and social care services in Northern Ireland (UK). The Southern HSC Trust serves a population of over 400 pregnant women with diabetes every year. The aim of this study was to evaluate the service provided by this Trust, in relation to the management of diabetes in pregnancy, and to investigate the willingness of women to adapt home monitoring during pregnancy.

## Methods

This study was conducted at the joint metabolic and obstetric antenatal clinic within the Craigavon Area Hospital, part of the Southern HSC Trust. The evaluation of the service by pregnant women with diabetes undergoing standard diabetes care was performed over a 4-week period. The participants were randomly selected from the hospital based metabolic-obstetric outpatient clinic. The study protocol, questionnaire and patient information leaflet were approved by the Research Manager, Consultants and the Heads of Services at the Southern HSC Trust. This study was carried out as Service Evaluation and the questionnaire was clearly entitled as “Service Evaluation – Improving Services for the Management of Diabetes in Pregnancy”. The documentation (Information for the Participants and Questionnaire) about the Service Evaluation was available at clinics for those interested to voluntarily and anonymously complete. Consent was Implied Consent in that those who completed the questionnaire did so voluntarily and anonymously on the basis of the information provided with the questionnaire. As this study was carried out as Service Evaluation it did not require ethical approval or research governance but the approval of the Heads of the Departments facilitated by the Research Manager at the Southern Health and Social Care Trust. This is according to the National Research Ethics Service Guidelines produced by the NHS Health Research Authority [10].

### Data collection: questionnaire

The questionnaire design (Supplementary file) was based on the previous work conducted by several of the authors (JG, CJW, JOD) to evaluate mHealth solutions. The questionnaire was completed in between 5 and 10 min and contained closed questions suitable for service evaluation purposes. The questionnaire consisted of 24 questions, including demographic descriptors, questions designed to determine time spent attending the antenatal clinics, information on the use of smartphones or tablets to manage health, acceptability of home monitoring of various parameters during pregnancy and remote management by the healthcare staff.

### Data analysis

The questionnaires were manually entered onto the Survey Monkey website, US. The data was analysed using the software integrated in the website.

## Results

Seventy women were approached to take part in the study. Seven women refused due to language barriers, sixty-two women completed the questionnaire independently and one participant was assisted by a clinic interpreter.

Participants’ demographics and time spent on current management of diabetes are presented in Table 1. The vast majority of the participants were white Caucasian between 25 and 35 years of age (69.8%), had one or more children (65.1%), had GDM (68.2%) and spent over 2 h attending the clinics (63.9%); 44.5% travelled for more than 30 min each way to the hospital.

**Table 1:** Participants’ demographics and time spent on current management of diabetes

	(%)	Responses (*n*)
Age		
15–20	0	0
20–25	11.1	7
25–30	25.4	16
30–35	44.4	28
35–40	14.3	9
40–45	4.8	3
The number of children		
None	34.9	22
1	30.2	19
2 or more	34.9	22
Education level		
None	0	0
GCSE (school exit level)s	22.2	14
A levels/AS level (further education)	22.2	14
Diploma	25.4	16
Degree	30.2	19
PhD	0	0
Type of diabetes		
Type 1 diabetes	22.2	14
Type 2 diabetes	3.2	2
Gestational Diabetes	68.2	43
Other	1.6	1
Not sure	4.8	3
Mode of transport to the diabetes pregnancy clinic		
Car	90.5	57
Bus	4.8	3
Train	1.6	1
Walk	0	0
Other	3.2	2
Length of time it takes to reach the clinic		
Less than 15 min	22.2	14
15–30 min	33.3	21
30–60 min	39.7	25
More than 60 min	4.8	3
Average waiting time in the clinic before first seeing any of the maternity or diabetes team		
Less than 15 min	11.1	7
15–30 min	49.2	31
30–60 min	25.4	16
More than 60 min	14.3	9
Average time spent in the clinic		
Less than 30 min	3.3	2
30 min −1 h	4.9	3
1–2 h	27.9	17
2–3 h	50.8	31
Greater than 3 h	13.1	8
Time typically spent on capturing each blood glucose measurement using the current paper based method every day		
Less than 10 min	71.2	42
10–20 min	22	13
20–30 min	0	0
More than 30 min	6.8	4
Frequency of recording data in blood sugar monitoring diary		
Each time you monitor a blood sugar level	59.3	35
At the end of the day	28.8	17
At weekends	1.7	1
At the end of the week	11.9	7
Prior to clinic	6.8	4

Smartphones and tablets are widely used among the participants; 93.5% of women were current users of smartphones, with almost an even distribution between iOS and Android users. The average time spent on using these mobile devices was between 1 and 5 h per day (73.3%). Over 70% of women using smartphones were already using the phones for health-related activities. However, only 8.8% used the smartphones to manage diabetes or health. Interestingly, around 40% of women did not record their blood glucose results immediately after measuring their blood glucose levels. Depending on what they would be required to input and test, between 78 and 90% of women found it acceptable to use a smartphone for managing their health in pregnancy. Most of the participants had no concerns about using smartphones or tablets in the context of managing their health or pregnancy (75.8%). More than 80% of women felt that it is acceptable to measure different clinical and lifestyle parameters related to their health at home and more than 60% of them are willing to discuss the results with clinic staff remotely. However, 91% of the participants would like to receive video, written, or face-to-face training. Further information from this survey is presented in Table 2.

**Table 2:** Details of the self-management of diabetes in pregnancy

	(%)	Responses (*n*)
Current usage of the smartphones		
Yes	93.5	95
No	6.4	4
Usage of the smartphones or tablets to obtain or manage health or pregnancy related information		
Yes	72.9	43
No	27.1	16
Health related activities that participants use their device(s) for		
Electronic communication (email, chat, text messages) with my doctor or midwife	45.6	26
Electronic communication with patient groups (group text messages/ Facebook/WhatsApp etc.)	29.8	17
Electronic communication with other health care providers	14	8
Finding doctors	3.5	2
Finding pharmacies	5.3	3
Managing my health data (blood sugar, heart rate, blood pressure)	8.8	5
For obtaining information about symptoms and conditions	66.7	38
For obtaining information about diagnostic and therapeutic procedures	31.6	18
For finding my own diagnosis	26.3	15
For documenting and managing fitness related data (e.g. using blood glucose meters, Fitbit etc.)	19.3	11
Other	14	8
Concerns in relation to using smartphones/tablets in the context of managing their health or pregnancy		
None	75.8	47
Concerns about data security	17.7	11
Concerns about the technical reliability of the devices	6.4	4
Concerns about the reliability of the software	9.7	6
The devices are too complicated to use	1.6	1
Not being able to attend as many hospital appointments/other concerns	3.2	2
Participants willingness to manage diabetes remotely		
Yes	66.7	42
No	14.3	9
Not sure	19	12

## Discussion

This study clearly demonstrates that there is a need for more efficient management of diabetes in pregnancy. The majority of women taking part in this study spent more than 2 h every 1–2 weeks at the joint metabolic and obstetric antenatal clinic; this excludes the time spent getting to and from the clinic. According to the National Institute for Clinical Excellence (NICE) guidelines, women with diabetes in pregnancy are required to attend joint diabetic and obstetric clinics every 1–2 weeks from conception (T1DM and T2DM) or from GDM diagnosis (around week 24–28) for assessment of blood glucose control and foetal growth [11]. Good glycaemic control in pregnant women with diabetes is key and it reduces complications associated with this condition in pregnancy [12–15]. The evidence that remote monitoring using digital technologies is acceptable to pregnant women and superior to the standard of care is limited [16–19]. A recent study using a remote blood glucose monitoring system demonstrated safety, user satisfaction and superior data capture. However, no differences in the maternal glucose management were observed, whereas pre-term births and caesarean deliveries were less common in the intervention group using a mobile-phone-based real-time blood glucose management system [20]. There have been no health economic assessments of this approach compared to usual care which are important to consider in the future due to unprecedented increase in the number of women with diabetes in pregnancy [7]. In a separate study, one of the authors (CO) conducted a virtual clinic at the same joint metabolic and obstetric antenatal clinic for pregnant women with diabetes who did not require frequent obstetrics appointments. This method identified that, on average 6–8 women (10%) were suitable candidates for remote monitoring. These women are likely to be diagnosed with GDM rather than more complex pregnancies in the presence of T1DM or T2DM. In our study, most of the women had GDM (68.2%), therefore, our findings are more relevant to this population of pregnant women. The growing number of women with diabetes in pregnancy, particularly GDM, is a significant burden for the healthcare systems globally. Considering pregnant women are generally motivated to self-monitor their condition remotely and are smartphone literate, digital technology for home monitoring provides an option for this cohort of women. Nevertheless, glucose management is often influenced by the obstetrics advice based on the foetal growth, which if restricted, in some cases requires admission to the hospital for steroid treatment. These obstetrics requirements exclude certain pregnant women for being suitable candidates for home monitoring. As a result, identifying pregnant women who can be safely monitored from home is a challenge that is influenced by obstetrics’ need.

The need for real-time data collection is also clear from this study. Indeed, only 60% of women record their blood glucose results at the time of monitoring (Table 1). Further studies are required to determine the clinical effectiveness of home monitoring interventions. Nonetheless, a systematic review of mobile phone-based interventions with clinical feedback showed that they improve glycaemic control (HbA1c) compared to standard care or other non-mHealth approaches by as much as 0.8% for T2M patients and 0.3% for T1D patients over the short-term (≤12 months) [21]. This suggests a role for these devices during pregnancy. The main limitation of our study is the small sample size. Nevertheless our findings confirm the general consensus amongst the healthcare professionals and patients regarding the significant strain on specialist diabetic maternity services, which often results in a poor patient experience. This area of important research has not been quantified and reported until now.

In this study, it was clear that women were not only willing to manage blood glucose remotely but that they were willing to monitor other health indicators during pregnancy. Most of women do not have any concerns about using digital technology to manage their health in pregnancy and are willing to have video or telephone conversations with the clinicians. The advantage of capturing the data remotely and in real-time is that it may increase data accuracy and reduces the time commitment and stress of frequent clinical appointments.

## Conclusions

Our service evaluation demonstrates that the self-management and home monitoring clearly appeals to pregnant women with diabetes and provides a good insight into the antenatal care provided in the UK. This is the first report that evaluates joint metabolic and obstetric antenatal care from the patients’ perspective, particularly focusing on the management of GDM. This study also confirms the long waiting times at antenatal clinics and willingness of this highly motivated group of patients to self-manage their condition with remote support by the healthcare staff.

## Data Availability

The datasets used and/or analysed during the current study are available from the corresponding author on reasonable request.

## Abbreviations

GDM: gestational diabetes mellitus
T1DM: type 1 diabetes mellitus
T2DM: type 2 diabetes mellitus
WHO: world health organisation
